# The effectiveness of the Screening Inventory of Psychosocial Problems (SIPP) in cancer patients treated with radiotherapy: design of a cluster randomised controlled trial

**DOI:** 10.1186/1471-2407-9-177

**Published:** 2009-06-09

**Authors:** Anna PBM Braeken, Lilian Lechner, Francis CJM van Gils, Ruud MA Houben, Daniëlle Eekers, Ton Ambergen, Gertrudis IJM Kempen

**Affiliations:** 1 Maastricht University, Faculty of Health, Medicine and Life Sciences, Department of Health Care and Nursing Science, School for Public Health and Primary Care (CAPHRI), Maastricht, The Netherlands; 2 Netherlands Open University, Faculty of Psychology, Heerlen, The Netherlands; 3 Maastricht Radiation Oncology, GROW Research Institute, Maastricht University, Maastricht, The Netherlands; 4 Institute Verbeeten, Radiation Oncology, Tilburg, The Netherlands; 5 Maastricht University, Faculty of Health, Medicine and Life Sciences, Department of Methodology & Statistics, Maastricht, The Netherlands

## Abstract

**Background:**

The Screening Inventory of Psychosocial Problems (SIPP) is a short, validated self-reported questionnaire to identify psychosocial problems in Dutch cancer patients. The one-page 24-item questionnaire assesses physical complaints, psychological complaints and social and sexual problems. Very little is known about the effects of using the SIPP in consultation settings. Our study aims are to test the hypotheses that using the SIPP (a) may contribute to adequate referral to relevant psychosocial caregivers, (b) should facilitate communication between radiotherapists and cancer patients about psychosocial distress and (c) may prevent underdiagnosis of early symptoms reflecting psychosocial problems. This paper presents the design of a cluster randomised controlled trial (CRCT) evaluating the effectiveness of using the SIPP in cancer patients treated with radiotherapy.

**Methods/Design:**

A CRCT is developed using a Solomon four-group design (two intervention and two control groups) to evaluate the effects of using the SIPP. Radiotherapists, instead of cancer patients, are randomly allocated to the experimental or control groups. Within these groups, all included cancer patients are randomised into two subgroups: with and without pre-measurement. Self-reported assessments are conducted at four times: a pre-test at baseline before the first consultation and a post-test directly following the first consultation, and three and 12 months after baseline measurement. The primary outcome measures are the number and types of referrals of cancer patients with psychosocial problems to relevant (psychosocial) caregivers. The secondary outcome measures are patients' satisfaction with the radiotherapist-patient communication, psychosocial distress and quality of life. Furthermore, a process evaluation will be carried out. Data of the effect-evaluation will be analysed according to the intention-to-treat principle and data regarding the types of referrals to health care providers and patient satisfaction about the with radiotherapists will be analysed by means of descriptive techniques. The process evaluation data will also be analysed by means of descriptive techniques.

**Discussion:**

Using the SIPP may prevent underdiagnosis of early symptoms reflecting psychosocial problems, should facilitate communication between physicians and patients about psychosocial distress and may contribute to adequate referral to relevant (psychosocial) caregivers.

**Trial Registration:**

NCT00859768

## Background

Cancer is a leading cause of death worldwide, accountable for 7.9 million deaths (around 13% of all deaths) in 2007 [1]. Cancer and its treatment may lead to psychosocial distress involving symptoms of depression and anxiety, and turmoil in the lives of patients and their families [2,3]. Patients entering radiotherapy treatment (RT) suffer from specific distress such as fear of RT and its side effects [4]. The prevalence rates of psychosocial distress in cancer patients as reported in numerous studies vary from 5 to 53%, depending on the study population and the method of distress assessment [3,5-10].

Nevertheless, psychosocial distress in cancer patients is often underdiagnosed by medical staff in oncology settings [11,12]. Recognition and treatment of psychosocial distress in cancer patients is crucial [12,13]. Even when psychosocial distress is on a sub-clinical level, it should be managed to prevent further deterioration to psychiatric disorders like major depression or adjustment disorder [10]. Psychiatric disorders affect many oncology outcomes, such as compliance with therapy and quality of life [12]. Effective treatment of psychosocial distress may affect the course of the disease and improve patients' quality of life [13,14]. Therefore, it is important to identify and treat cancer patients with psychosocial distress at an early stage.

Validated screening instruments are useful tools to recognise psychosocial distress in cancer patients [2]. Besides recognition of psychosocial distress, self-reported questionnaires have proven to be good instruments to facilitate communication between patients and physicians with respect to psychosocial distress [12,15]. Although a number of well-validated screening instruments exist, including the Hospital Anxiety and Depression Scale [16], the Brief Symptom Inventory [5] and the General Health Questionnaire [17], these instruments require time and effort in administering and scoring, which may prevent their systematic use in clinical oncology settings [7,18,19]. Feasible screening instruments should meet the following criteria: be very brief, preferably fitting on one page[20]; be easy to complete (to avoid further distress by excessive questioning) [21]; be easy to score and interpret by medical staff [22]; and facilitate communication between physicians and patients about psychosocial distress [12,15]. The Screening Inventory Psychosocial Problems (SIPP) is a valid and reliable Dutch questionnaire (see methods section). The SIPP was specifically developed for use in Dutch cancer patients and for measuring a variety of distress symptoms [23].

Although the SIPP is being used in several hospitals and health care facilities in the Netherlands, there is still little known about the effects of using this questionnaire in consultation settings [23]. Therefore, we performed a study to assess the effectiveness of the SIPP in a clinical oncology setting. This paper presents the design of a cluster randomised controlled trial (CRCT) evaluating the effectiveness of using the SIPP in Dutch patients with the most common cancer types treated with radiotherapy.

### Aims

Primary aims are to study the effect of the SIPP on the number and types of referrals of cancer patients with psychosocial problems to psychosocial caregivers. Secondary aims are to study the effects of the SIPP on: 1) patients' satisfaction with the radiotherapist-patient communication during first consultation, 2) psychological distress in both the short- and long-term, and 3) quality of life in both the short- and long-term. Additionally, a process evaluation will be performed. The aim of the process evaluation is to gain insight into factors potentially influencing the effectiveness of the SIPP and factors facilitating future implementation of the SIPP in oncology care settings, if the SIPP proves to be effective.

## Methods/Design

### Study Design

**Figure 1:**
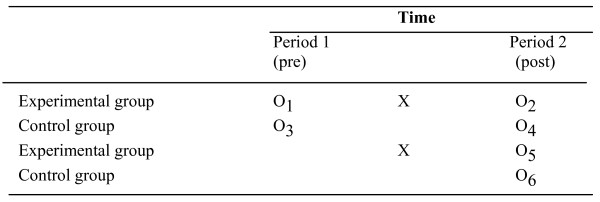
**General description of the Solomon four-group design **[53]. O: Observation. X: Intervention.

The design of this study is a CRCT because it is less prone to contamination bias [24-26]. In addition, we used a Solomon four-group design. The Solomon four-group design is an experimental design with two experimental groups and two control groups (Figure 1). Pre-test measures are used for one experimental and one control group. Following exposure of both experimental groups to the intervention, post-test measures are assessed in all four groups.

### Intervention

The SIPP is a short, valid and reliable 24-item self-reported questionnaire that systematically identifies psychosocial problems in Dutch cancer patients. The psychometric properties of the SIPP were studied in an as yet unpublished study (submitted, copy available upon request). This questionnaire was originally developed in the Netherlands by Pruyn and colleagues in 1997 (then named the Integral Checklist) [23], and was further adapted in several Dutch pilot studies [20,27]. The SIPP assesses:

• physical complaints (seven items, score range 0–14),

• psychological complaints (10 items, score range 0–20),

• social and financial problems (four items, score range 0–8), and

• sexual problems (three items, score range 0–6 with an additional option of "not applicable" (N/A)).

Items are rated on a three-point scale of 0 (no) to 2 (yes). Higher scores indicate poorer functioning.

Before the start of the study, the radiotherapists in the experimental condition are trained in using and interpreting the SIPP. According to the intervention procedure, the patient receives the SIPP at two different time points during their RT period: just before the first consultation with the radiotherapist (one to three weeks before starting RT) and before the last consultation with the radiotherapist at the end of the RT period (five to nine weeks after completing the first SIPP). At both times, the completed SIPP is handed to the radiotherapist at the start of the consultation. The radiotherapist checks the scores of the SIPP to get an overview of potential psychosocial problems and the patient's needs for psychosocial care. Psychosocial problems are discussed with the patient during the consultation. Referral to a psychosocial caregiver at the Institute Verbeeten in Tilburg occurs only with the permission of the patient.

### Care as usual

Patients in the control group receive care as usual. No recent guidelines for the systematic assessment of psychosocial problems in cancer patients exist at the Institute Verbeeten. In the control group, radiotherapists may refer patients to psychosocial caregivers (social workers) at the Institute Verbeeten. However, this occurs according to the radiotherapist's judgment about the presence or absence of psychosocial problems in patients. Referrals to psychosocial caregivers at the Institute Verbeeten will be registered in both the patient's records and by the psychosocial caregivers.

### Recruitment of the study population

Recruitment of cancer patients takes place at the Institute Verbeeten, a radiation oncology department in Tilburg, the Netherlands. Only cancer patients receiving RT are eligible for this study.

The following inclusion criteria are used: 1) patients with the most common cancer types such as lung, prostate, bladder, rectum, breast, cervix, endometrial, skin carcinoma and Non-Hodgkin; 2) patients 18 years of age or older and 3) patients with no metastases. Exclusion criteria are: 1) patients receiving palliative treatment, ≤ 10 fractions of RT; 2) patients unable to read and speak Dutch and 3) patients unable to complete questionnaires. Patients who meet the inclusion criteria are sent information about the study. Those willing to participate in the study are asked to sign a consent form and are only allowed to participate after completing and returning it.

### Randomisation and stratification

**Figure 2:**
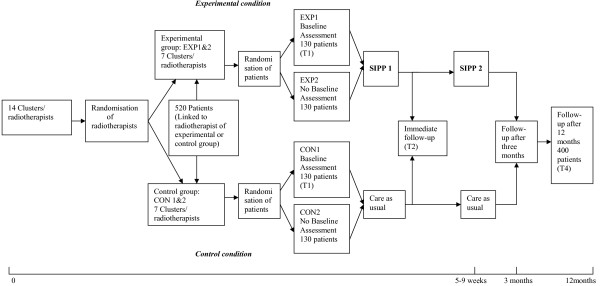
**Cluster randomised control trial – Solomon four-group design**.

To reduce contamination bias, radiotherapists, instead of patients, are randomly allocated to the experimental or control groups. First, the radiotherapists are stratified according to the general percentages of incoming patients they referred to a (psychosocial) caregiver in the period 2006–2007. Within their stratum, they are either randomly assigned to the experimental or to the control condition (see Figure 2). Thus, in both conditions there are equal numbers of radiotherapists who previously referred relatively more and less cancer patients to (psychosocial) caregivers. Patients are linked to their radiotherapist. Therefore, they are randomised to the experimental condition with or without pre-measurement or to the control condition with or without pre-measurement through their radiotherapist (see Figure 2). Patients who visit their radiotherapists in odd weeks are assigned to the experimental/control condition with a pre-measurement and patients who visit their radiotherapists in even weeks are assigned to the experimental/control condition without a pre-measurement. Radiotherapists of the experimental condition are asked not to discuss this study with their colleagues of the control condition.

### Sample size and power analysis

This study is powered on the primary outcome measures, i.e. the effect of the SIPP on the number and types of referrals of patients with psychosocial problems to relevant (psychosocial) caregivers. Sample size calculation shows that 92 patients per condition are required to compare the number of referred patients to (psychosocial) caregivers between conditions with a 80% power and a significance level of 0.05 (one-sided) [28]. This calculation is based on results reported by Pruyn and colleagues [23]. In their study, the use of the Integral Checklist resulted in more referrals from 2 to 11%. This means that for our study a total of 368 patients have to be included for the analyses.

The consequence of adopting a CRCT is that the outcome for each patient can no longer be assumed as independent [26]. Such a lack of independency has implications for study size and power [29]. The standard sample size has to be inflated to take account of the cluster design by using an estimate of the Intra Cluster Correlation [30,25,26,31]. For our study, no appropriate ICC was available. Therefore, as in the CRCT of Cumming and colleagues [32], we assume an ICC of 0.005 as appropriate because the variance of our outcome measurements between clusters can be considered low compared to the variability between the patients within a cluster. To retain power, the standard sample size should be multiplied by the design effect (d, d = 1+ρ (m-1)), where m is the size of a cluster unit and ρ the ICC [25,33]. We therefore multiplied our standard sample size with a design effect (d) of 1.13. With an expected dropout rate of 20%, the corrected sample size for the CRCT is determined at 520.

It is to be noted that stratification by baseline value of the outcome variable decreases the sample size required [31]. In our study, radiotherapists are stratified according to the general percentages of incoming patients they referred to a (psychosocial) caregiver. Stratification of this primary outcome variable reduces the between-cluster variability in the primary outcome. In a study by Elridge and colleagues the sample size required decreases of almost 50% by using stratification of the primary outcome [31]. To be on the safe side regarding the power of our study we decided not to correct for stratification of the radiotherapists. Recruitment and data collection is anticipated to continue for 24 months.

### Measures

#### Outcome measures

Table 1 presents the primary and secondary outcome measurements and time of assessment.

**Table 1:** Primary and secondary outcome measures of the effect evaluation

Variables	No. of items	T1	T2	T3	T4
**Primary outcome measures:**					
Number and types of referrals	8/NA	-	-	Q	Q/DR
**Secondary outcome measures:**					
Patients' satisfaction with the radiotherapist-patient communication	5	-	Q	-	-
Extent of psychological symptoms:					
Symptoms of anxiety and depression (HADS)	14	Q	-	Q	Q
General psychological distress (GHQ-12)	12	Q	-	Q	Q
Quality of life (EORTC QLQ-C30)	30	Q	-	Q	Q
**Additional variables:**					
Socio-demographic variables	4	-	Q	-	-
Medical variables	4	-	-	-	DR

T1: Before first consultation (baseline, only groups with pre-measurement); T2: After consultation;

T3: Three months after baseline; T4: 12 months after baseline; N/A: not applicable; Q: questionnaire:

DR: data record.

HADS: Hospital Anxiety and Depression Scale [16]; GHQ-12: General Health Questionnaire-12 items [17];

EORTC QLQ-C30: European Organisation for Research and Treatment of Cancer,

Quality of life Questionnaire 30 items [46].

#### Primary outcome measures

The primary outcome measures are the number and types of referrals of patients with psychosocial problems to psychosocial workers at the Institute Verbeeten and/or to external health care providers (e.g. psychologists, psychiatrists). The number and types of referrals to (psychosocial) caregivers are measured at three months (T3) and 12 months (T4) after baseline assessment with a self-developed questionnaire by the patient and from registration records of the psychosocial caregivers at the Institute Verbeeten. This questionnaire comprises eight items on whether the patient is referred to a psychosocial caregiver and the types of problems the patient has experienced (e.g. financial, psychological, and sexual). The psychosocial caregivers at the Institute Verbeeten collect data from all referred patients, including types of problems, number of sessions needed and further referral to an external (psychosocial) caregiver.

#### Secondary outcome measures

The secondary outcomes are classified in three dimensions: 1) patients' satisfaction with the radiotherapist-patient communication during the first consultation; 2) extent of psychological symptoms at T3 and T4; and 3) patients' quality of life at T3 and T4.

*Patients' satisfaction with the radiotherapist-patient communication *is measured directly after the first consultation with the radiotherapist (T2) by the patient completing a self-developed questionnaire comprising five items. The first four items measure whether physical complaints, psychological complaints and social and sexual problems were discussed with the radiotherapist (item range 1 (yes) to 3 (no) plus "not applicable"). The fifth item measures the patient's general opinion (item range 1 (very bad) to 6 (very good)) about the communication with the radiotherapist during the first consultation.

*The extent of psychological symptoms *is measured with the Hospital Anxiety and Depression Scale (HADS) [16] and the Goldberg's General Health Questionnaire-12 item version (GHQ-12) [17]. Patients complete these self-reported questionnaires at baseline and at T3 and T4.

The HADS is a valid, reliable and useful instrument [16] that is widely used in studies among cancer patients [34-40]. It is considered unbiased by coexisting general medical conditions because its questions do not refer to somatic symptoms associated with depression [41]. The HADS consists of a brief subscale of anxiety and a subscale of depression. Both subscales comprise seven items. Ratings by patients are made on four-point scales (0–3, with 3 indicating greatest distress). Higher scores on each subscale indicate a greater presence of problems [16,42,43].

The GHQ-12 is a well-validated instrument [17] that has been used in numerous studies among cancer patients [34,36,44,45]. It is a standardised measure of psychiatric morbidity across a wide range of patients [43]. It was intended for use in general practice settings as a screening instrument for detecting verifiable psychiatric morbidity (generally anxiety and depression). It measures "usual state" rather than chronic (long-term) problems [43]. The GHQ assesses with 12 items whether the patient considers him- or herself better, the same, worse or much worse over the previous four weeks than he/she "usually" is. Those who indicate that their symptoms are unchanged or have decreased receive a score of 0, while those who report that their symptoms have increased receive a score of 1. Total scores range from 0 to 12 [17].

*Quality of life *is measured using the European Organisation for Research and Treatment of Cancer, Quality of life Questionnaire (EORTC QLQ-C30) [46]. The EORTC QLQ-C30 is validated and widely used [30,47-50]. It is a 30-item cancer-specific measure that assesses health-related quality of life. The EORTC QLQ-C30 comprises:

• five functional subscales: physical (five items), role (two items), emotional (four items), cognitive (two items) and social functioning (two items);

• a subscale about global health status and quality of life during the past week (two items);

• three symptom scales: nausea/vomiting (two items), fatigue (three items) and pain (two items); and

• six single items measuring appetite loss, insomnia, constipation, diarrhoea, dyspnoea and financial difficulties due to illness or treatment.

The items on the five functional scales and three symptom scales have four-point response choices of 1 (not at all), 2 (a little), 3 (quite a bit) and 4 (very much). Categories 3 and 4 are regarded as indicators of clinically-significant symptom levels. Both items of the subscale about global health status and quality of life use a seven-point visual analogue scale (VAS) ranging from 1 (very poor) to 7 (excellent) [43]. Patients completed the self-reported questionnaire EORTC QLQ-C30 at baseline and at T3 and T4.

#### Additional variables

Socio-demographic and medical variables are gathered to provide insight into the characteristics of the sample and to interpret the outcomes of the study.

*Socio-demographic variables *age, gender, marital status and educational level are assessed directly after the first consultation with the radiotherapist (T2).

*Medical variables *before RT include cancer site, adjuvant chemo-treatment, TNM-classification and Karnofsky Performance Index (KPI). The TNM-classification is a systematic way of describing the size, location and spread of a tumour. Once established, it must remain unchanged in the patient's record because the definitive TNM-classification is determined just before either initiation of treatment or making the decision not to treat [51]. The KPI emphasises physical performance and dependency. The scale is weighted towards physical dimensions of quality of life rather than social and psychological dimensions. Patients are assigned to categories by a physician. The KPI takes no account of the patient's feelings. The KPI varies from 100 (normal, no complaints, no evidence of disease) to 0 (dead) [43]. Medical data are extracted from patient records.

#### Process evaluation

The aim of the process evaluation is to gain insight into factors potentially influencing the effectiveness of using the SIPP and factors facilitating future implementation of the SIPP. Process evaluation data are collected using self-developed questionnaires completed by patients of the intervention groups directly after their first consultation with the radiotherapist and by radiotherapists directly after the first consultation and twice during the total recruitment period of patients. Table 2 provides a short overview of process evaluation measures. The process evaluation outcome measures are:

**Table 2:** Outcome measures of the process evaluation

Variables	No. of items	T1	T2	T3	T4
Radiotherapists' opinion about the usefulness of the SIPP in general	9	-	-	-	Q
Radiotherapists' opinion about the usefulness of the SIPP after first consultation	4	-	Q	-	-
Radiotherapists' suggestions for improvement of the intervention or other remarks	2	-	-	-	Q
Patients' opinion about the usefulness of the SIPP after first consultation	3	-	Q	-	-
Patients' suggestions for improvement of the intervention or other remarks	2	-	Q	-	-

T1: Before first consultation (baseline, only groups with pre-measurement); T2: After consultation;

T3: Three months after baseline; T4: 12 months after baseline; Q = questionnaire.

• nine items on the opinion of radiotherapists about the usefulness of the SIPP in general, e.g. "Did you find discussing the SIPP with the patient useful?" (item range 0 (not useful) to 10 (very useful));

• four items on the opinion of radiotherapists about the usefulness of the SIPP after each consultation with a specific patient, e.g. "How much extra time (in minutes) does discussing the results of the SIPP take?";

• opinion of the patients about the usefulness of the SIPP after the first consultation, e.g. "Did you find discussing the SIPP with the radiotherapist useful?" (item range 0 (not useful) to 10 (very useful); and

• two open questions for suggestions for improvement of the intervention or other remarks.

### Statistical analysis

Descriptive techniques will be used to describe patients' background characteristics. To detect potential differences between the experimental and control groups at the start of the study, the baseline levels of psychological distress and quality of life will be compared. Furthermore, to identify potential differences between the two experimental groups and the two control groups (both with and without pre-measurement) at T3 the extent of psychological distress and quality of life will be compared. If these latter differences emerge, the four groups need to be handled separately in the analyses, i.e. the experimental and control groups with pre-measurement and the experimental and control groups without pre-measurement will be compared separately. If not, the two experimental groups and the two control groups will be combined in one group.

Data of the effect evaluation will be analysed according to the intention-to-treat principle. Univariate, multivariate and descriptive techniques are applied to estimate the effect of the intervention by comparing the experimental with the control groups with regard to the primary and secondary outcomes at the follow-up measurements and by comparing the pre-test (T1) with post-tests (T3 and T4). Potential confounding factors and baseline differences will be checked and included in the model if necessary. Since dependency between outcome variables from the same cluster may exist, as well as between repeated measurements within patients, multilevel modelling will be carried out. Furthermore, multilevel modelling minimizes the loss of data through dropout by including all available data from participants in the analyses. Dropout will be described.

The process evaluation data (collected from radiotherapists and patients of the experimental groups) will be analysed by means of descriptive techniques.

### Ethics

The Medical Ethics Committee of the Twee Steden Hospital in Tilburg, the Netherlands, granted approval for conducting this study.

The protocol is registered in the ClinicalTrials.gov register number NCT00859768.

## Discussion

This study will provide insight into the actual systematic effects of using the SIPP in consultation settings. It is important to know the possible effects of using the SIPP on aspects such as communication between physicians and patients, early recognition and treatment of psychosocial problems, the extent and severity of experienced psychological problems among cancer patients and the quality of life of patients over a long period. Until now, there have been no RCTs on the effectiveness of the SIPP on aspects such as quality of life among cancer patients and the extent of psychological distress that cancer patients experience over a long period after the initial radiation or other treatment of the cancer. Until now, there have also been no reports on the differences in number of patients receiving psychosocial help because of using the SIPP.

The design of this study is a CRCT. Cluster randomisation is applied because randomisation at the patient level may jeopardise the validity of the study since patients of the experimental and control conditions may visit the same radiotherapist for consultation. Furthermore, radiotherapists may alter their communication style, attitude or may pay more attention to patients' psychosocial distress because of using the SIPP. The Solomon four-group design is chosen in order to check for potential pre-measurement effects on intervention outcomes [52,53].

In this study, we decided to include patients with the most common cancer types and with a reasonably good prognosis because patients were asked to complete questionnaires 12 months after baseline measurement.

One limitation of this study is that the results can not be generalised to all Dutch cancer patients since our study population consisted of cancer patients that received RT. Further studies outside the radiotherapy setting would be required to generalise the results.

### Future implementation

Using the SIPP may prevent underdiagnosis of early symptoms reflecting psychosocial problems and may contribute to adequate referrals to psychosocial caregivers. Therefore, using the SIPP may lead to a reduction of psychological problems and a better quality of life among Dutch cancer patients in both the short- and long-term. If the SIPP proves to be effective, the results of this study may help motivate physicians to use the SIPP as a standard method for early detection of psychosocial problems in oncology departments in the Netherlands and abroad.

### Progress of the study

Recruitment of eligible patients commenced in April 2008 and will end in July 2009, resulting in 520 eligible patients being included in the study. The follow-up period will continue until July 2010. Results will be published in relevant journals.

## Competing interests

The authors declare that they have no competing interests.

## Authors' contributions

LL and GK obtained funding together with FG, RH, DE and AB. LL, GK, FG, RH, and AB were involved in conception and design of the study. AB is the investigator and works under supervision of LL and GK. LL, GK and AB designed some questionnaires. RH and TA were advisors in the statistical analysis plan. AB and DE coordinated the entry and validation of the data. AB drafted the manuscript, with input from the other authors. All authors mentioned in the manuscript read and approved the final version.

## Pre-publication history

The pre-publication history for this paper can be accessed here:

http://www.biomedcentral.com/1471-2407/9/177/prepub
